# Environmental Factors That Influence Stem Cell Migration: An “Electric Field”

**DOI:** 10.1155/2017/4276927

**Published:** 2017-05-15

**Authors:** Stephanie N. Iwasa, Robart Babona-Pilipos, Cindi M. Morshead

**Affiliations:** ^1^Institute of Biomaterials and Biomedical Engineering, University of Toronto, Toronto, ON, Canada M5S 3E1; ^2^Toronto Rehabilitation Institute, University Health Network, Toronto, ON, Canada M4G 3V9; ^3^Department of Surgery, University of Toronto, Toronto, ON, Canada M5S 3E1

## Abstract

*Environmental Stimulus of Electric Fields on Stem Cell Migration.* The movement of cells in response to electric potential gradients is called galvanotaxis. In vivo galvanotaxis, powered by endogenous electric fields (EFs), plays a critical role during development and wound healing. This review aims to provide a perspective on how stem cells transduce EFs into directed migration and an understanding of the current literature relating to the mechanisms by which cells sense and transduce EFs. We will comment on potential EF-based regenerative medicine therapeutics.

## 1. Introduction

Stem cells reside in restricted microenvironments where their development and behaviour are controlled by a variety of cues. Signalling from the niche and microenvironment controls many aspects of stem cell behaviour including regulation of quiescence versus proliferation, as well as regulating modes of division and migration. The environmental cues that activate signalling pathways are often subtle and can vary in homeostatic versus pathological conditions.

Electric fields (EFs) are a physical cue inherent in physiological environments. They are typically created when charged particles (ions) are separated across cell membranes thereby creating transmembrane electric potential differences or across sheets of coupled cells in epithelium thereby creating transepithelial potential differences [1]. When an injury occurs, transepithelial potentials are disrupted. For instance, a skin wound can disrupt the insulating cell barrier thus creating a potential of zero volts at the injury site. A new electric potential difference is then created between the injury site and the regions surrounding the wound. This EF is significantly greater near the wound edge, dropping off with distance from the wound [2]. Depending on species, location of injury (skin, cornea), distance from the wound edge, and time after injury, the strength of the EF can vary extensively from around 0.6 to 200 mV/mm [3–5]. These injury-generated potentials are critical for directing the migration of cells at the wound margin towards the injury site because disrupting the EF prevents wound closure [6–7]. Similarly, EFs are critical for tissue development. These EFs can range from 10 to 20 mV/mm as is found beneath the neural plate ectoderm, to larger EFs of 1000 mV/mm across the neural tube [8, 9]. The removal or reversal of these in vivo EFs causes developmental defects such as tail abnormalities and malformed limbs [9–11]. Hence, EFs provide an important environmental cue that regulates cell behaviour during development and following injury.

For many years, it has been recognized that EFs are dominant cues that guide the persistent migration of many different cell types. Many cell populations from mammalian, amphibian, and fish species have been identified to undergo galvanotaxis: the directional migration of cells in an EF. These cells include neural crest cells [12], somatic epithelial cells of the cornea [13–15], lens [16], and retina [17], vascular endothelial cells [18], Schwann cells [19], leukocytes [20], macrophages [21], keratinocytes [22, 23], osteoblasts and osteoclasts [24], chondrocytes [25], and fibroblasts [26]. In addition, stem cells and their progeny from the central nervous system (forebrain and spinal cord-derived) [27, 28] and human mesenchymal stem cells [29, 30] as well as human embryonic and induced pluripotent stem cells [31] undergo galvanotaxis. The direct current EF intensities that are used to induce in vitro migration of different cell types and ages of organisms (i.e., embryonic to adult) vary considerably ranging from as low as 3 mV/mm to greater than 1000 mV/mm [19, 21]. This range of EFs is comparable to those found in vivo during development and wound healing [3, 8].

## 2. Neural Stem Cells and Endogenous Electric Fields

While a variety of cells are responsive to EFs during development and in adulthood, stem cells are of particular interest due to their regenerative potential. Regulating the behaviour of tissue-specific stem cells has garnered much attention. Tissue-specific stem cells are self-renewing and multipotent with their progeny restricted to generating cells specific to their tissue of origin. Understanding the cues that regulate the behaviour of tissue-specific stem cells and their progeny is a priority for regenerative medicine.

Neural stem cells reside in the germinal zone of the developing central nervous system and are found in the periventricular regions of the adult nervous system [32, 33]. The forebrain subependyma lining the lateral ventricles is a neurogenic region that contains the largest population of neural stem cells in adulthood. In vitro, individual neural stem cells isolated from the subependyma can proliferate to form clonally derived clusters of cells termed “neurospheres” in the presence of mitogens (epidermal growth factor and fibroblast growth factor). A neurosphere consists of a pure population of stem cells and their progeny (together termed neural precursor cells) (NPCs) [32, 33]. There is great interest in harnessing the regenerative potential of these cells to promote neural repair. One method to control NPC migration is through EFs.

EFs in neural tissue are normally present due to neural oscillations and display complex patterns. The effects of these endogenous EFs on cell migration are mostly unknown. Interestingly, under physiological conditions in the adult brain, the rostral migratory stream is a well-defined pathway where ongoing neurogenesis and NPC migration occurs towards the olfactory bulb. Along this pathway, a small 3 mV/mm EF is present and it is speculated that this endogenous EF may play a role in the in vivo directed migration of NPCs [34]. Cao et al. demonstrated that reversing the field in ex vivo tissue slices resulted in reversed direction of migration [34]. However, this has not been explicitly tested by disrupting the EF in vivo. Endogenous EFs are also generated as a result of injury. Following an ischemic insult, there is prolonged cellular depolarization which causes a drop in extracellular direct current potential [35–36]. This change in potential could contribute to directing NPC migration to the site of injury as is observed following cortical stroke [37–39]. These findings are of particular interest when considering whether one could use EFs to enhance endogenous NPC cell migration to injury sites as a means to enhance neural repair.

Notably, EFs have also been shown to promote axon outgrowth, increasing the length and directing the growth of axons from embryonic chick explants and *Xenopus* embryonic neurons [40–42]. Furthermore, a number of neural cell types have been demonstrated to undergo galvanotaxis including oligodendrocyte precursors [43], astrocytes [44], and neuronal cells [45]. The reader is directed to reviews done by Yao et al. where nervous system cell migration in direct current EFs has been discussed in more detail [46, 47]. In this review, we will focus on the role of EFs in regulating cell migration of neural stem and progenitor cells.

## 3. Cell Mechanisms for Sensing and Transducing Electric Fields

Cells undergoing galvanotaxis have speed, velocity, and directionality. Speed is a measure of how fast the cells are migrating. Velocity is a measure of how fast the cells are migrating towards the cathode or anode. Finally, directionality is a measure of the cells' straight line path towards the cathode or the anode. These components of galvanotaxis can be decoupled, that is, change in directedness without changing velocity or speed, highlighting the complexity of the mechanisms involved in galvanotaxis [27, 48]. Although there are many unknowns related to the cellular mechanisms that underlie galvanotaxis, it is well established that ion channels are critical for sensing and transducing EFs. In response to EFs, both intracellular molecules polarize and channels themselves polarize. In turn, this affects the normal ion flow through ion channels causing polarized intracellular response [48, 49]. The polarized response can then trigger cytoskeletal changes directing the cell's migration.

### 3.1. Neural Stem Cells

Adult-derived NPCs respond to EFs by migrating towards the cathode. This EF-induced migration is specific to undifferentiated NPCs, and they respond within 15 minutes of application of a 250 mV/mm EF. Differentiated progeny of NPCs (neurons, astrocytes, and oligodendrocytes derived from neurospheres in the presence of fetal bovine serum) do not migrate in the presence of an EF [27]. Interestingly, the directed migration of undifferentiated NPCs is regulated, in part, by epidermal growth factor (EGF). In the absence of EGF, undifferentiated NPCs have reduced directedness and velocity. Indeed, in the presence of the EGFR antagonist erlotinib, a significant loss in NPC migration velocity was observed [27]. Meng et al. suggested that EGFR is redistributed in the cell membrane in response to EF application [28]. Further, EGFR downstream effector phosphoinositide 3-kinase (PI3K) has key roles in actin polymerization. Phosphatidylinositol-3,4,5-trisphosphate (PIP3) and actin were increased at the leading cathodal edge. Thus, EGFR senses the EF while the activation of the PI3K pathway, which could include Rho GTPases as reviewed by Hanna and El-Sibai, enables actin polymerization at the leading edge thereby contributing to propelling the cell forward during galvanotaxis [50]. Notably, calcium is also required for actin polymerization and the extension of processes at the leading edge of migrating cells [51], and perhaps not surprisingly, galvanotaxis of NPCs is in part regulated by calcium. Buffering extracellular calcium in an EF of 115 mV/mm for 1.5 hours prevents NPC migration [52]. Thus, the polarization of actin and the influx of calcium contribute to persistent cell migration towards the cathode.

Further highlighting the importance of receptors, Li et al. showed that *N-*methyl-*D*-aspartate receptors (NMDARs) are important in explant cultures of embryonic germinal zone from the developing brain [53]. When placed in the presence of an EF, a subpopulation of cells in the explant cultures undergoes cathodal migration. In addition, NMDAR downstream pathways of Rac1 activator Tiam1, phosphorylated p21-activated kinase 1, and actin were upregulated in response to the EF. Moreover, application of an NMDAR antagonist significantly decreased cell migration to the cathode and inhibited the increased associations between NMDAR and its downstream pathways. Since the explant cultures contained a mixed population of stem cells, progenitor cells and mature neural cells, it is not possible to determine which receptor and/or intracellular signalling pathway was specific (or not) to the EF-induced migration of stem versus somatic cells [53]. Despite this caveat, the study reveals that multiple receptors and intracellular signalling pathways are involved in transducing EFs to NPC migration.

Another cue that plays a role in the EF-induced migration of NPCs is cell-matrix/cell-cell interactions. Cao et al. examined pure populations of adult-derived NPCs and cells from a human neuroblastoma cell line and found increased expression of the cell adhesion proteins N-cadherin and *β*-catenin in the presence of EFs [54]. There was a concomitant upregulation of the P2Y purinoreceptor in the presence of the EF, and interestingly, blocking the receptor using drugs or siRNA resulted in a loss of directedness in NPCS [54]. To note, cell-cell contact has been demonstrated to have effects in altering the sensitivity of epithelial cells to EFs. Clustered epithelial cells respond to lower EFs than single cells though they required more time in the presence of the EF before aligning [55]. Furthermore, fibroblasts have shown different galvanotactic behaviour depending on extracellular matrix molecules as their migration changes with different collagen I substrate concentrations [26]. Hence, cell-matrix and cell-cell interactions appear to play a role in galvanotaxis as well.

### 3.2. Nonneural Stem Cells

The galvanotactic response of a variety of nonneural stem cell populations has been explored. In vitro studies support the idea that the galvanotactic response of cells varies depending on the passage number of the cells. For instance, passage 1 mesenchymal stem cells migrated towards the anode while more extensively passaged cells (passage 3-4) that were in culture for longer periods migrated towards the cathode. One possible explanation for this finding is that the mesenchymal stem cells were differentiating into a more mature chondrogenic phenotype with continued passaging, and chondrocytes have been shown to migrate towards the cathode [56]. Zhao et al. demonstrated that human bone marrow-derived mesenchymal stem cells will migrate towards the anode but the migration speed and direction is reduced with higher passage number [30]. These findings reinforce the fact that cultured cells, outside of their in vivo niche, can change their behaviour depending on environmental cues.

Most interestingly, the galvanotactic response of human-derived mesenchymal stem cells was shown to be donor dependent [29]. Bone marrow-derived mesenchymal stem cells from three independent donors showed cathodal migration. However, their migration speeds, displacement, and time delay between application of EF and onset of migration were all different. This work highlights the fact that optimization of EF application is critical for potential application in vivo.

### 3.3. Somatic Cells

Much of the work analyzing how cells sense and transduce EFs into migratory behaviours has been done on somatic cells. It is likely that the galvanotactic response of stem cells and somatic cells share some similarities; however, this is yet to be established.

Nakajima et al. performed a large-scale screening for ion transporter expression in human cells during galvanotaxis [48]. The importance of inwardly rectifying potassium channel Kir4.2 was established in a number of cell lines including human corneal epithelial cells (cathode-migrating cells), immortalized human keratinocytes (anode-migrating cells), and a human breast adenocarcinoma line (anode-migrating cells). They demonstrated that Kir4.2 knockdown via siRNA interfered with galvanotaxis in each of these cell types in EFs up to 500 mV/mm whereby the breast adenocarcinoma cells displayed reduced migration speed whereas the corneal epithelial cells and keratinocyte migration speed was not changed. This highlighted the differences in mechanisms depending on cell type and the fact that it was independent of whether galvanotaxis was anodally versus cathodally directed. The proposed mechanisms through which this channel senses the EF is through the polarization of polyamines in the cell. There was no obvious polarization of Kir4.2 channels in the presence of EFs; however; the intracellular polyamines which bind to the Kir4.2 channel were polarized to the cathode in both cathode- and anode-migrating cells [48]. This asymmetric distribution affects how the polyamines bind to the Kir4.2 channel which can cause local changes in membrane potential, osmolality, and ionic environment which in turn is important for galvanotaxis. Similar to NPCs, PIP3 is involved in galvanotaxis of human corneal epithelial cells, immortalized human keratinocytes, and a human breast adenocarcinoma line. Knocking down the EF-sensing Kir4.2 inhibited PIP3 from polarizing to the leading edge of the process during galvanotaxis [48]. As previously discussed, this protein is involved in actin polymerization whereby the polymerization of actin could push the cell forward at the leading edge.

A number of other channels have been shown to play a role in galvanotaxis. Yang et al. showed that the epithelial sodium channel (ENaC), a heterotrimeric channel which mediates transepithelial sodium transport and water balance in polarized epithelia, plays a role in the directionality of galvanotaxis in keratinocytes [49]. Epithelial keratinocytes from an *α*ENaC (the major pore-forming subunit of the channel) knockout mouse and human keratinocytes with an ENaC siRNA-mediated knockdown migrated with similar or increased speed compared to wild-type controls. However, their migration was undirected in the presence of an EF. Of interest, they found that a human lung epithelial cell line that did not undergo galvanotaxis underwent directed migration in the presence of an EF when ENaC was overexpressed. Furthermore, unlike the Kir4.2 channels, ENaC polarized to the cathode side of keratinocytes in the presence of an EF. The knockout of ENaC prevented the formation of stable lammellipodial protrusions on the leading edge of keratinocytes in the presence of an EF [49]. Thus, ENaC senses EFs and can contribute to galvanotaxis through lamellipodia. This highlights two different pathways by which channels can sense EFs: through the polarization of small molecules that bind to the channel and/or through the polarization of the channels themselves.

Similar to its importance in NPC migration, Trollinger et al. found that EF application in conditions that decreased calcium influx (using strontium, a calcium substitute) resulted in a significant decrease in the directionality (94% decrease), and to a lesser extent, a reduction in speed (33% decrease) for human keratinocytes [57]. Hence, calcium is important in the galvanotactic migratory response of both stem and somatic cells.

A review by Funk discussed how mammalian cells sense and transduce EFs through calcium influx and Na(+)/H(+) exchangers (NHE3) [58]. The galvanotaxis of cathode-directed osteoblasts, anode-directed osteosarcoma cells, and cathode-directed HEK293 cells were compared and contrasted. Both osteoblast cells and osteosarcoma cells had a detectable increase in intracellular calcium levels initiated in the trailing edge in the presence of an EF [59]. This increase in calcium level could activate the protein kinase C (PKC) pathway either directly or through phosphatidylinositol 4,5-bisphosphate (PIP2), whose expression was also modified during EF exposure, and second messenger signalling lipid diacylglycerol [60–62]. PKC was shown to contribute to the patchy localization of NHE3 which colocalized with *β*-actin at the leading edge membrane protrusions [61, 62]. Furthermore, *γ*-tubulin complexes interacted with phosphorylated NHE3 in patches in the leading edge of the cell [61]. Indeed, the *γ*-tubulin was thought to aid in establishing the microtubule polarity within the cell as they were observed at the microtubule-organizing centres which could help orient the cell in the presence of persistent cues such as EFs [63].

Although there are known mechanisms through which cells sense and transduce EFs into migration, many questions persist. The different signalling pathways that regulate cell migration to the cathode or anode still remain unsolved. While both osteoblasts (cathode-directed) and osteosarcoma (anode-directed) cells express similar channels such as NHE3, NHE1, and Na, K-ATPase (NaKA), they are differentially activated in the presence of EFs and their inhibition has different effects on their migratory behaviour [60]. Further, intracellular and extracellular pH has also been shown to be important. The presence of H+ clouds at the leading edges of cathodally directed osteoblast cell migration has been observed; however, this was not seen in anodally directed osteosarcoma cell migration. These findings are consistent with the idea that intracellular changes in pH may dictate the direction of migration [60]. Reducing extracellular pH can lead to a complete reversal of the direction of migration, causing human keratinocytes to migrate to the anode instead of undergoing cathodal migration as is seen under physiological pH conditions [64]. Additionally, cells respond to EFs in a dose-dependent manner. Bovine epithelial cells migrate in opposite directions depending on the value of the EF. They migrate towards the anode between 150 and 200 mV/mm and towards the cathode at 50 mV/mm [16]. Thus considering the environment and the strength of the EF cues will be critically important for promoting directed cell migration.

## 4. Clinical Application of Electrical Stimulation for Therapeutic Interventions

Electrical stimulation therapies are currently used in the clinic and have been implemented to promote wound healing of chronic ulcers, albeit with limited success [65]. Transcranial direct current electrical stimulation (tDCS) and deep brain stimulation (DBS) are in clinical practice for neural stimulation of a variety of disorders including mood disorders such as depression, epilepsy, hypokinetic movement disorders, and psychiatric diseases [66, 67]. The cellular mechanisms underlying the success of these strategies are not well established. For tDCS, it is suggested that the success of the treatment relates to the orientation of the neurons being activated, NMDA sensitivity for neuroplasticity, intracortical neurotransmitter concentrations, changes in transmembrane proteins, or changes in cortical connectivity and/or spinal connectivity as recently reviewed by Roche et al. [68]. The optimal stimulation parameters in humans have not yet been clearly established. Although for tDCS, <2.5 mA is considered the “standard,” higher stimulation has been employed in some studies [69].

For DBS, a range of frequencies, pulse widths, and amplitude values as well as the differences between constant-current and constant-voltage have been reported [70, 71]. The location of the implanted electrodes and the disorder being treated play a large role in determining the best stimulation parameters for successful treatment [70, 71]. It is proposed that DBS mimics a lesion thereby leading to inhibition of local neuronal networks, disruption of abnormal firing in the brain, and/or it could also involve stimulation-induced release of neurotransmitters locally and through larger networks as reviewed by Chiken et al. and Herrington et al. [72, 73]. DBS also promotes synaptic plasticity and network reorganization which could explain the slower recovery responses observed following DBS [73]. In terms of cell-specific effects, it has been proposed that astrocytes play a key role in DBS therapies by regulating neuronal activity and contributing to long-term potentiation and depression [74].

There is significantly less research on the effects of these clinical stimulation parameters on NPCs; however, the evidence that exists supports the hypothesis that NPCs are affected. In the case of tDCS, Rueger et al. performed 10 days of transcranial stimulation and examined cell migration 3 days after the last stimulation [75]. They reported that cathodal tDCS (but not anodal) increased the number of endogenous NPCs within the cortex (outside their normal in vivo subependyma niche) [75]. One interpretation is that the NPCs migrated towards the cathode, similar to what is seen in vitro. In a more recent separate study, Keuters et al. transplanted fluorescently labelled and iron oxide-labelled NPCs into the striatum and the corpus callosum of rats and used the same stimulation parameters as Rueger et al. (15 minutes per day at 500 *μ*A for 10 days) [75, 76]. They demonstrated that anodal tDCS increased the migration of transplanted NPCs within the striatum and on the corpus callosum in the rat brain. The stimulation did not lead to directed migration, but instead, the cells were dispersed more widely in the stimulated brain [76]. These findings suggest that EF induced a more rapid migration of cells within the brain parenchyma while the lack of optimization of the stimulus parameters could account for the undirected migration. Electrical stimulation can also affect the differentiation profile of NPCs. In vitro, electrical stimulation of NPCs enhances differentiation towards neurons, although, immediately after stimulation of NPCs remain undifferentiated [27, 52, 77]. In vivo, there has been reported enhanced neurogenesis with significant increases in doublecortin-positive neuroblasts in the subependyma of the adult rodent forebrain at 2 days post-tDCS [78]. Further, a separate study supported the activation of NPCs with DBS, reporting increased proliferation in the neurogenic dentate gyrus of the rodent brain [79]. Finally, increased cell proliferation has been observed in the human brains of Parkinson's patients that received DBS, compared to normal and untreated Parkinson's brains [80]. Taken together, these studies suggest that electrical stimulation used in current clinical therapies can activate NPCs.

Based on the ability of electric stimulation to activate NPCs, we propose that electrical stimulation paradigms could be developed to stimulate NPC migration and attract more stem cells to injury sites. Accordingly, to translate the in vitro galvanotaxis studies that direct stem cell migration to the clinic, it is important to consider the use of clinically relevant waveforms for EF delivery. To this end, Babona-Pilipos et al. demonstrated, for the first time, that a balanced biphasic monopolar EF at 400 Hz was able to direct NPC migration in vitro [77]. This is an exciting step forward as the use of charge-balanced stimulation would reduce electrochemical reactions that could create toxic by-products through degradation of the implanted electrode, which would necessarily occur if direct current EFs were applied [81]. Other negative effects of overstimulation of neural tissue include enhanced inflammatory reactions including the activation of endogenous microglia [81]. The careful use of EF stimulation for neural repair could harness the ability of EFs to direct cell migration to injury sites and contribute to the goal of wound healing in the nervous system.

## 5. Conclusions

**Figure 1:**
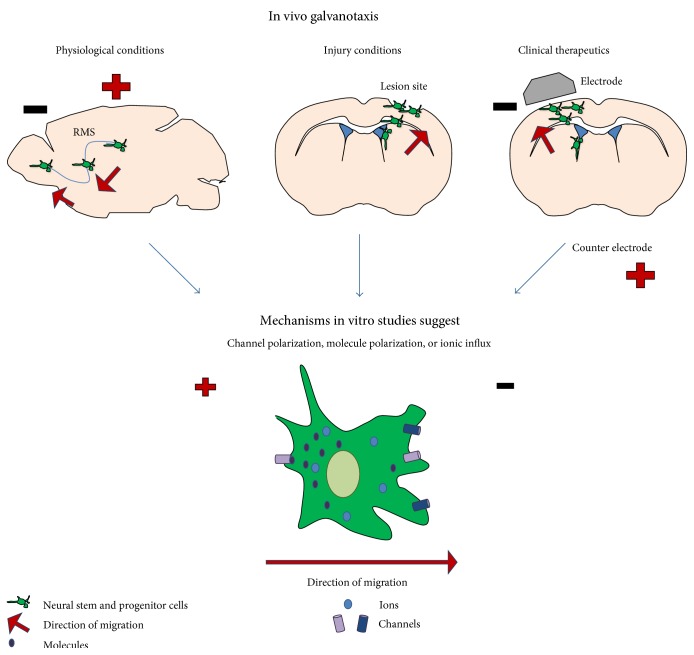
Overview of how electric fields affect stem cells in vivo and how the electric fields can be sensed. Sagittal brain section featured in the top left, coronal brain sections featured in the top middle and right. RMS: rostral migratory stream.

Much work has been done to examine the galvanotactic response of somatic cells and stem cell populations; however, a thorough understanding of how EFs direct cell behaviour is not well established. Work to date has revealed that EFs activate a number of channels and that variations in the extracellular and intracellular environment as well as the distribution of channels on the membrane contribute to the galvanotactic response (Figure 1). Cell-specific factors such as cell type and differentiation state, along with time in culture, cell-matrix, and cell–cell contact can influence the galvanotactic response of cells. Further insight into the underlying factors and mechanisms involved in cell migration in response to EFs will enhance the application of EFs in regenerative strategies.
